# Leveraging the strengths of mice, human stem cells, and organoids to model pancreas development and diabetes

**DOI:** 10.3389/fendo.2022.1042611

**Published:** 2022-10-21

**Authors:** David S. Lorberbaum, Dylan Sarbaugh, Lori Sussel

**Affiliations:** Barbara Davis Center for Diabetes, University of Colorado Anschutz Medical Campus, Aurora, CO, United States

**Keywords:** hPSC-derived beta cells, mice, organoids, diabetes, pancreas development, GATA6

## Abstract

Diabetes is an epidemic with increasing incidence across the world. Most individuals who are afflicted by this disease have type 2 diabetes, but there are many who suffer from type 1, an autoimmune disorder. Both types of diabetes have complex genetic underpinnings that are further complicated by epigenetic and environmental factors. A less prevalent and often under diagnosed subset of diabetes cases are characterized by single genetic mutations and include Maturity Onset Diabetes of the Young (MODY) and Neonatal Diabetes Mellitus (NDM). While the mode of action and courses of treatment for all forms of diabetes are distinct, the diseases all eventually result in the dysfunction and/or death of the pancreatic β cell - the body’s source of insulin. With loss of β cell function, blood glucose homeostasis is disrupted, and life-threatening complications arise. In this review, we focus on how model systems provide substantial insights into understanding β cell biology to inform our understanding of all forms of diabetes. The strengths and weaknesses of animal, hPSC derived β-like cell, and organoid models are considered along with discussion of GATA6, a critical transcription factor frequently implicated in pancreatic dysfunction with developmental origins; experimental studies of GATA6 have highlighted the advantages and disadvantages of how each of these model systems can be used to inform our understanding of β cell specification and function in health and disease.

## Introduction

Diabetes is an extremely prevalent disease afflicting more than 10.5% of the world’s population in 2021, with statistics indicating that number will increase to over 12% by 2045 (1). The costs associated with diabetes treatment also continue to rise, with current estimates upwards of $966 billion, likely to increase to over $1 trillion in the coming years. One reason for the staggering monetary costs associated with diabetes is that in the absence of a cure, there is a life-long requirement to rigorously monitor one’s health. In the case of T1D for example, exogenous insulin must be continuously administered, either by regular injections or with an insulin pump, to manage blood glucose homeostasis. This careful regulation prevents life threatening complications that can result from hypo- or hyperglycemia but requires constant management. Despite the challenging healthcare and economic costs associated with diabetes, and the substantial effort dedicated to understanding this disease, a cure is still elusive. To make progress towards a cure, our understanding of the molecular mechanisms of diabetes must be clarified.

While each type of diabetes is associated with different etiologies, all ultimately result in the death and/or dysfunction of the pancreatic insulin-producing β cell. The two most common forms of the disease are known as type 2 and type 1 diabetes (T2D, T1D). T2D is predominantly associated with insulin resistance. This occurs when peripheral tissues – such as adipose, liver, and muscle – are unable to respond appropriately to insulin signaling leading to increased blood glucose levels. Eventually, overworked β cells become exhausted and dysfunctional, disrupting insulin production and further contributing to hyperglycemia. Interestingly, however, not all individuals with T2D have insulin resistance. This phenomenon is most common in Asian populations who generally are lean but have dysfunctional β cells that are unable to appropriately regulate blood glucose levels (2, 3). In all cases, prolonged hyperglycemic conditions can lead to life altering conditions including retinopathy, neuropathy, cardiovascular disease, chronic kidney disease, or stroke. Lifestyle, environment, and genetics are some of the main contributors to T2D. Treatments range from improving one’s diet and exercise routine to medications like Metformin to increase insulin uptake by peripheral tissues and reduce glucose production in the liver or Sulfonylureas which can increase insulin release. Depending on the underlying causes and severity, T2D can be effectively managed to reduce or prevent complications.

Type 1 diabetes (T1D) is a distinct disease caused by an autoimmune attack on β cells. The first stage of T1D is usually characterized by the presence of autoantibodies, which erroneously attack β cells, leading to the reduction of β cell mass. Up to 15% of people diagnosed with T1D, however, do not have autoantibodies, suggesting a nonimmune source for β cell dysfunction. These patients are generally excluded from many of the clinical trials discussed in this review but could provide critical insights into our understanding of T1D (4). However, in both idiopathic or autoantibody positive cases of T1D, β cell loss will lead to hyperglycemia in the second stage of the disease, followed by presentation of clinical symptoms, such as polyuria, weight loss, and excessive thirst caused by increasingly severe hyperglycemia in stage three. As in T2D, prolonged hyperglycemia leads to life-threatening complications. T1D frequently impacts children but can also arise in adults later in life. There is a stronger genetic component to T1D as compared to T2D, with upwards of 60 genetic loci being highly correlated with development of the disease, recently reviewed in (5). Interestingly, there are also many people with T1D associated risk alleles, but who never develop the disease or develop it much later in life as compared to others who share that same allele. When considering the increased incidence of T1D across the world, it is likely that there are also environmental factors associated with T1D.

A third, less common type of diabetes can arise from single gene deficiencies, and therefore are characterized as monogenic diabetes. One form of monogenic diabetes is known as Maturity Onset Diabetes of the Young, or MODY. MODY is characterized by the loss of β cell function and is frequently misdiagnosed as T1D because of the overt phenotypic similarities between the two diseases, including affecting younger, lean patients. However, MODY does not involve autoimmune destruction of β cells but arises from single gene mutations that disrupt the development and function of β cells either directly or indirectly, impairing their ability to produce and secrete insulin. These mutations often directly affect β cell genes, but can also impact the acinar tissue surrounding pancreatic islets eventually leading to β cell dysfunction, as in the case of MODY8 (6). The inability of β cells to function properly ultimately leads to hyperglycemia and downstream complications. Another form of monogenic diabetes is known as Neonatal Diabetes Mellitus (NDM). While much less common than MODY, these cases are also often confused with T1D because they are generally diagnosed in infants, but again do not involve autoimmune deficiencies. NDM is usually found as part of a broader syndrome and can be transient, naturally reversing at some point after diagnosis, although many of the cases are permanent and require tailored treatment depending on the molecular origins of the disease (7). The advent of less expensive and more widely available whole genome sequencing provides better tools to identify the full complement of mutations involved in the underlying genetics of monogenic diabetes. These technologies have demonstrated that many forms of monogenic diabetes are caused by β cell transcription factor (TF) mutations (8–12). TFs are used repeatedly throughout development in multiple contexts to regulate gene expression and modulate cellular function. When particular domains or regulatory elements harbor mutations, this can affect their function in both specifying cells and maintaining function into adulthood (13). The inherent nature of studying TF mutations is complicated because of their repeated use, often requiring a variety of context or temporally specific proteins or environmental signals.

While there are numerous transcription factors that have been implicated in diabetes, we focus on GATA6 because this gene has been identified as a cause of several pancreas-related diseases, including NDM and T2D, but there are still many open questions about how it functions due to conflicting results in different model systems, and often even within the same models. GATA6 is an essential TF for β cell specification and numerous other developmental processes (14, 15). Highlighting the dynamic roles of GATA6 in multiple different contexts, there have been nearly 80 heterozygous GATA6 patient mutations identified and while the majority lead to cardiac and/or pancreas defects, there is extensive variability (16). More than 60% of patients with GATA6 mutations have pancreas defects that can lead to NDM, T2D, and/or pancreas agenesis, a condition in which the patient does not develop a pancreas or has a substantially smaller pancreas. All of these diseases can have life-threatening consequences and necessitate lifelong treatment. The reasons for the various outcomes associated with GATA6 mutations are not entirely understood. Elucidating mechanisms by which GATA6 regulates development and function will provide better care and more targeted therapies for patients.

While the variability associated with diabetes makes this disease difficult to model, defining the underpinning genetic mechanisms will improve treatment of all forms of diabetes. In this review we focus on the advantages and pitfalls of using mice, human pluripotent stem cells, and organoids to address these open questions about how to study the roles of β cell genes, using GATA6 as an example throughout. Ultimately, it will be necessary to use multiplatform approaches to unravel the undefined mechanisms of β cell specification and function.

## Mice and other animal models

Many animal models have been critically important for studying diabetes, largely because mechanisms of sugar processing and metabolism are highly conserved from invertebrates to mammals (17). Traditionally rats were used because of their large physical size, facilitating studies of physiology. This led to the creation of many rat models of T1D and T2D including the Bio-Breeding (BB), Long Evans Tokushima Lean (LETL), Komeda Diabetes Prone (KDP) and LEW-iddm rats for T1D and the Goto-Kakizaki (GK) and Zucker diabetic fatty (ZDF) rats for T2D (18, 19). These models have all provided exceptional insights into β cell biology and diabetes but, until recently, were not amenable to substantial genetic analyses. On the other hand, *Drosophila melanogaster*, or the fruit fly, does not have a pancreas but represents an excellent genetic model of metabolism and the signaling pathways regulating these processes; including the ability to secrete insulin like proteins (Ilps) in response to high levels of sugar (20, 21). *Danio rerio*, or zebrafish, have also provided important genetic models of pancreas development by virtue of their transparent bodies that allow the *in vivo* visualization of development (22–24). Zebrafish have also emerged as a useful genetic model for studying obesity and diabetes (16). Here, however, we focus on the specific strengths and weaknesses of using mice, which are currently the most common model of β cell development, function, and disease (25). In particular, researchers have extensively used mouse models to define the roadmap for pancreas development from primitive gut tube to β cells, which has become the source code for creating and fine tuning all stepwise stem cell and organoid differentiation protocols (26). Furthermore, numerous mouse models of diabetes have greatly extended our knowledge of pancreas function in health and disease.

**Figure 1:**
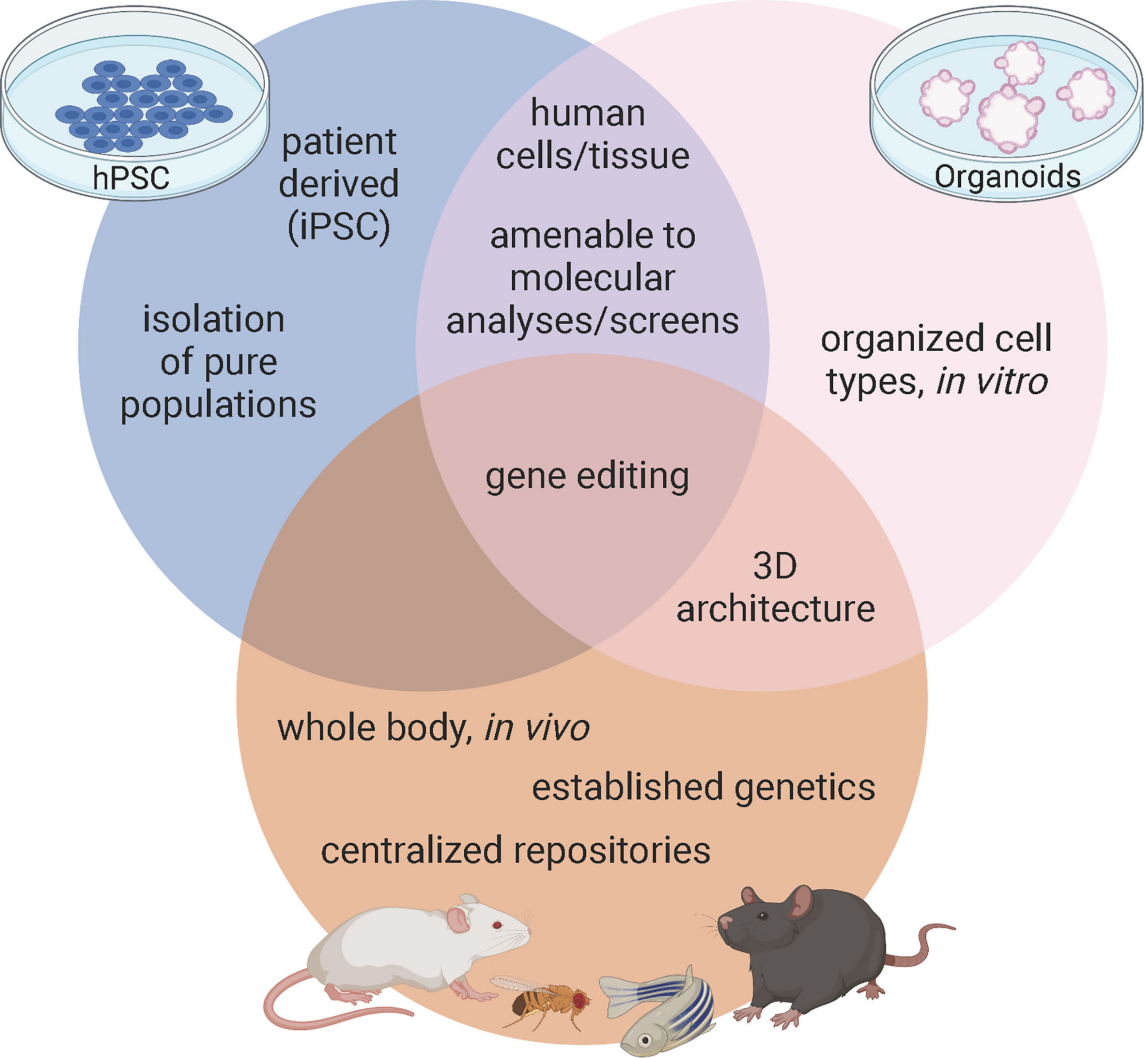
Advantages of model systems to study pancreas development and disease. Figure created with biorender.com.

A major strength of using mice in diabetes research stems from their established genetics and shared physiology with humans contained within an intact, manipulatable *in vivo* system (**Figure 1**). In pancreatic islets, β cells not only interact with each other but also interact with alpha, delta, and PP cells as well as the vasculature, nervous system, and immune cells. These cellular connections are integral to understanding β cell development, maturation, and function. While β cells have been the primary focus of diabetes research, both T1D and T2D etiologies are immensely complex and interactions with other endocrine cells and exocrine tissue in the pancreas can contribute to this disease. Furthermore, in models of T1D, the interaction between pancreatic islets and the immune system must be carefully considered, while research on T2D and insulin resistance needs to involve the interaction between the pancreatic islets and peripheral tissues.

Several mouse models have been created for the purpose of understanding the etiologies of diabetes, including the spontaneous autoimmune T1D (NOD), models that can genetically induce T2D (db/db), and mice that model polygenic or monogenic forms of the disease (19, 27, 28). Furthermore, researchers have been manipulating murine genomes for decades; initially by homologous recombination and more recently with CRISPR-Cas 9 precision editing. These techniques allow us to not only harness full body genetic knock outs and temporal, tissue, or cell type specific knockouts using recombination *via* Cre-Lox, but we can also model patient specific mutations like those associated with GATA6. With established techniques, strong genetic models, and central repositories of previously characterized mutants, mice provide researchers a valuable tool for defining how pancreatic β cells develop and function in health and disease (**Figure 1**).

**Figure 2:**
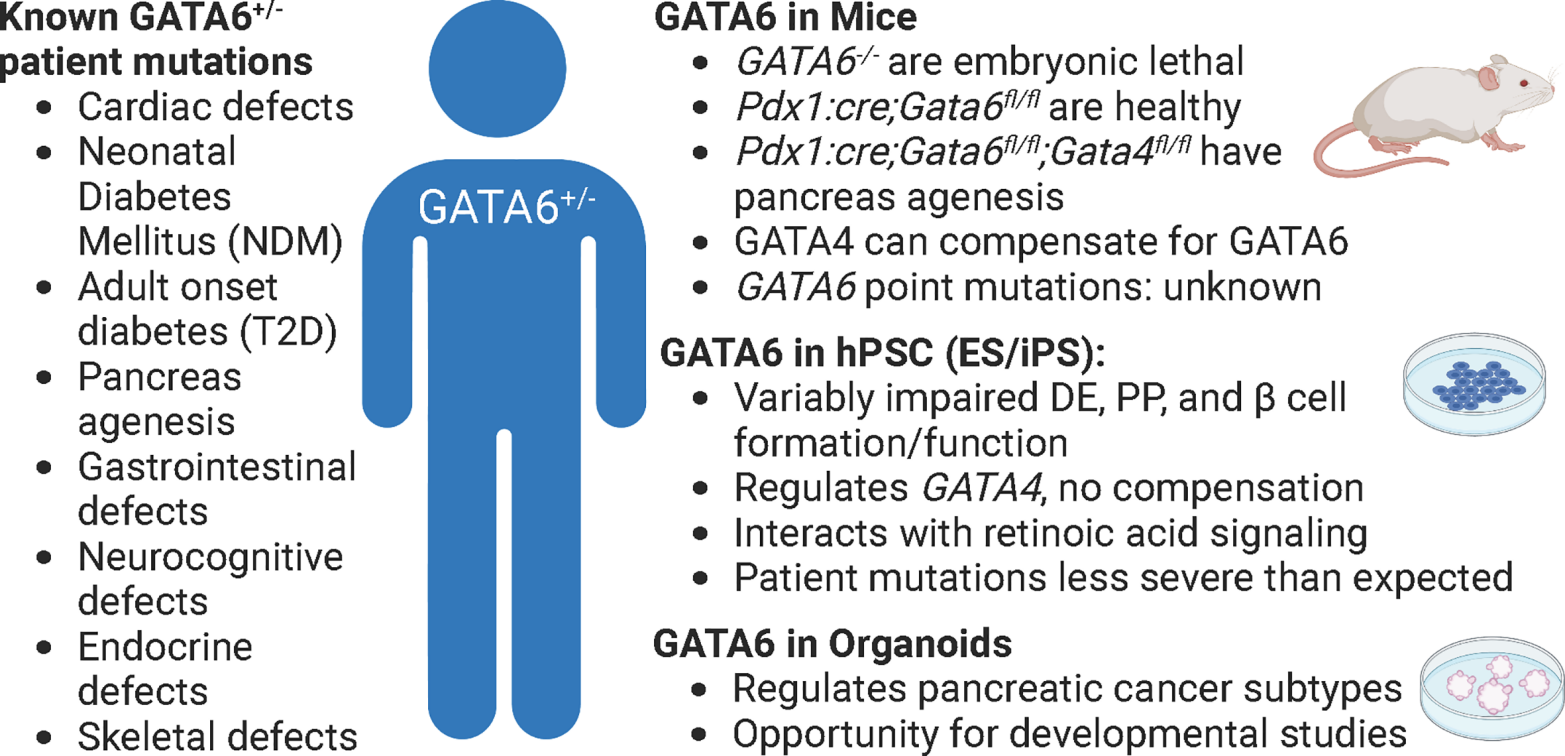
GATA6 heterozygous mutations in patients, mice, and human cells. The effects of GATA6 heterozygous mutations in patients have many detrimental outcomes (left), but these are difficult to study in any single model of pancreas development (phenotypes summarized by model, right). *Pdx1:cre; Gata6/4^fl/fl^* animals lack GATA6 in all pancreatic progenitors. Figure created with biorender.com.

Although mice afford the benefit of an intact physiological platform to study diabetes, there are several short comings to this model as well. First, while overall function of pancreatic islets are highly conserved between mice and humans, there are substantial differences in islet architecture and distribution of islet cell types. For example, murine islets consist of approximately 75% β cells that are clustered in the center of the islet and surrounded by a mantle of the other endocrine cell populations: the alpha cells (~20%), delta (~5%), and PP cells (<5%). However, in human islets, the endocrine cell populations are more interspersed and contain more even ratios of cell types: ~55% β, ~35% alpha, ~10% delta, and <2% PP cells (17, 29). While all of these cell types function similarly in mice and humans, it is important to keep these differences in mind when using mice as a model system. Second, while mice and humans share nearly all of the same genes, there are important differences in how many of these conserved genes function (30). Of particular interest, there are several instances of genetic haploinsufficiency leading to severe disease in humans that mouse models fail to recapitulate (31). One example lies with the GATA family of transcription factors. Heterozygous GATA6 mutations in humans have been identified as a major cause of pancreas agenesis that often results in diabetes (16, 32, 33). Defining the mechanisms by which heterozygous loss of GATA6 leads to developmental defects and β cell loss using mice, however, has been challenging. Whole body deletion of GATA6 is embryonic lethal (15), necessitating the creation of tissue specific knockouts – which is distinct from the human condition. Using floxed alleles, neither heterozygous nor homozygous pancreas specific knockouts of GATA6 substantially disrupt pancreas development. However, there are two GATA family members expressed during pancreas development: GATA6 and GATA4 (14) and simultaneous knockout of GATA6 and GATA4 in murine pancreas progenitors leads to complete agenesis at birth. These experiments uncovered substantial genetic redundancy in mice; losing either GATA6 or GATA4 alone results in minor pancreas phenotypes (34, 35) (**Figure 2**). The differences between GATA6 function in mice and human pancreas specification are still being defined and will likely include studies focused on interactions with other genes, signals, and/or environmental cues. A similar example of species-specific differences can be found in the transcription factor HNF1α. Heterozygous mutations in this gene lead to MODY3, the most common form of monogenic diabetes (36, 37) and while these mutations can be modeled using hPSC derived β cells (38) inducing similar mutations in mice does not affect β cell development or function (39). While both GATA6 and HNF1α are highly evolutionarily conserved, these examples highlight that there are still species-specific disease modifiers that need to be addressed. Third, though there are many mouse models available to study diabetes, mice are highly inbred and therefore lack genetic diversity as a population when compared to humans. This is especially important when considering the disease to be modeled. In this case, diabetes is an extremely genetically diverse disease in which the environment plays a significant role, making it harder to recapitulate in a mouse model. Finally, a more technical limitation of using *in vivo* models deals with collecting enough cellular material required for certain molecular techniques. While ultimately these limitations apply to all models, the scalability of hPSC derived cells, often achieved using bioreactors, provides an advantage that is not available to *in vivo* murine models. For example, collecting individual endocrine progenitor cell populations or the rarer islet cell populations to perform molecular experiments like ChIP-sequencing or mass spectrometry generally requires sorting and pooling cells from multiple animals, especially during embryonic stages. Newer technologies like CUT&RUN and single cell (sc) RNA-/ATAC-sequencing are making it easier to complete these important molecular experiments with fewer cells, however models like hPSCs and organoids can theoretically provide unlimited quantities of cells, often making these experiments more feasible.

## hPSC derived β-like cells

A more recently used model system to elucidate mechanisms of β cell specification and function are human pluripotent stem cells (hPSCs). This platform includes both human embryonic stem cells (hESCs) and induced pluripotent stem cells (hiPSCs), the latter of which can be derived directly from patient samples. Both types of cells can be directed towards a β cell fate through a series of stepwise addition or removal of signaling molecules that mimic *in vivo* development. hPSC derived β-like cells also hold the clinical promise of generating an unlimited supply of β cells to replace those lost in diabetes. While tremendous progress has been made in β cell differentiations, there are still several shortcomings with stem cell-derived β cells that will be needed to improve this as a model and potential therapeutic treatment for people with diabetes.

The most immediate benefit of using hPSC derived β-like cells as either a model or therapeutic tool is the fact that these are human cells and hold immediate relevance to understanding human β cell specification, function, and disease without relying on evolutionary conservation like in rodent models. While nearly all patients with T1D require exogenous insulin, either through injections or a pump, to regulate blood glucose levels, if fully functional and mature β cells could be generated in a dish, they could potentially be transplanted into patients to treat, or even cure diabetes with appropriate attention to immune responses (40). Many groups have demonstrated hPSC derived β-like cells’ ability to restore glucose homeostasis in diabetic murine models (41–43) with exciting advances in clinical trials as well. Two recent studies transplanted hPSC derived endocrine progenitor cells into small cohorts of patients with T1D (44, 45) and while none of the patients achieved true insulin independence, the implanted hPSC derived cells were able to secrete insulin in response to changes in blood glucose levels (46). This progress demonstrates the feasibility of hPSC derived β cells as a future therapeutic option for people with diabetes.

In addition to their clinical potential, hPSC derived β-like cells are highly amenable to genome editing using tools like ZFNs, TALENs, and CRISPR/Cas9 (47). With the advent of these highly targetable genome editing technologies, it is becoming routine to knock out genes or generate mutations in genes of interest in hPSCs to study gene function, similar to what has been done in rodents for decades using homologous recombination. Once the mutation is made, hPSCs can then be differentiated into β like cells alongside isogenic controls, creating a paired experiment in which wildtype and mutant samples are treated in parallel throughout the entirety of the differentiation, theoretically reducing variability. Experiments like these can be scaled up to generate a theoretically unlimited quantity of cells at different developmental stages to take advantage of molecular analyses, like mass spectrometry or ChIP-sequencing that generally require more cells than can be collected from *in vivo* based experiments in mice or donated human tissue. Many laboratories have taken advantage of this model to complete these kinds of studies; one particular tour de force was completed by Zhu et al. (48), in which eight critical β cell TFs that had known roles in murine pancreas specification but murkier, or even unknown, roles in human pancreas development were systematically mutated using CRISPR/Cas9 and examined during human β cell differentiations. Combining molecular and functional analyses, the authors were able to confirm the roles of these genes and identify new human specific functions as well. A similar approach can be used for more targeted approaches, as several studies examining GATA6 disease mutations have used hPSC derived β-like cells as a model (49–51). In these studies, the authors highlight another advantage of this system, by pairing these CRISPR/Cas9 edited hESC lines directly to patient derived iPSC lines. Their findings demonstrate a significant role for GATA6 in formation of definitive endoderm, β cell specification, and β cell function. The Gadue group also went on to use a combination of genome editing with the hPSC system to identify critical regulatory elements of GATA6 as a disease modifier, providing another example of the utility of hPSCs to define mechanisms of β cell function in health and disease (13).

An inherent limitation of hPSC derived β-like cells arise from the classic caveats associated with *in vitro* models of development: they are only as good as our knowledge of natural developmental processes. One important consideration concerns oxygen levels during stem cell maintenance and β cell differentiation (52). The amount of oxygen available to cells must be carefully controlled during β cell differentiation protocols to promote normal β cell specification by mimicking *in vivo* conditions (53). In addition, the proper nutrients available in media as well as biomechanical conditions are essential concerns when optimizing cultures for studies of cell fate determination (54, 55). While we have defined many stages of β cell differentiation through decades of work in mice, rats, and zebrafish (17, 56, 57), there remain numerous knowledge gaps to be addressed. Stepwise β cell differentiations require the precise inclusion and exclusion of signaling molecules, and even femto-molar differences in concentration of a particular agonist, can have drastic effects on differentiation (58). Furthermore, when using hPSC derived β cells as a model system, it is possible to overlook tissue interactions and extrinsic signaling factors that might play a significant role in the process. For example, while modeling a particularly severe heterozygous GATA6 mutation, the Gadue group found that iPSCs from a patient who suffered from severe neonatal diabetes and pancreas agenesis were able to be differentiated into pancreatic progenitors and even β-like cells, even though the patient suffered from severe agenesis (50, 59). Owing to the ability to easily examine the contribution of different molecules and signaling in the hPSC platform, the authors went on to determine that only under limiting amounts of retinoic acid, an experiment completed based on knowledge from animal models of development (60–62), did the heterozygous GATA6 mutation impair β cell differentiation.

Another limitation of hPSC derived β cells is the variability between protocols among different groups specializing in this technique. While the overall differentiation scheme is generally similar with common landmarks along the way (i.e. PDX1/NKX6.1 positive pancreatic progenitors), there are key differences in the duration of exposure to signals that are used. For example, when comparing three popular protocols (41, 63, 64), Wesolowska-Andersen and colleagues highlighted differences in key pathway agonists for bone morphogenetic protein signaling (BMP), epidermal growth factor (EGF), and protein kinase C (PKC), which are all known to be important for pancreatic endocrine specification (65). The authors went on to complete a series of whole genome analyses, including RNA- and ATAC-sequencing at each stage of differentiation to identify key overlapping features of the different protocols, but also found many significant differences at key stages. They also note that all protocols had to be amended from what was originally published, highlighting the dynamic nature and continuous tweaking of the protocols in the field (65).

## Organoids

Organoids are an emerging experimental model that leverages many benefits of mice and hPSC derived β like cells. These self-organizing 3D structures are grown in culture with the goal of recapitulating an organ of interest containing multiple functional cell types (66–68). Organoids can be established directly from pancreatic ducts by harnessing the proliferative power of progenitor cells (69–71) or by relying on proliferation from a disease state using donated tissues or biopsies from patients who suffered from pancreatic ductal carcinomas, for example (72). More recently, derivation of pancreatic organoids from hPSCs has emerged as a method that can take advantage of starting from a common progenitor and using standardized conditions to expand into any particular type of tissue (73). While the relatively young organoid field holds great promise as a model of pancreas development and function, as well as for precision medicine, there are still several pitfalls to consider.

Many advantages of pancreatic organoids overlap with those of hPSC derived β cells: they both provide the potential of unlimited sources of human derived cells, can be genetically modified with techniques like CRISPR, are amenable to chemical screens, and hold therapeutic potential to treat diabetes. In addition to these benefits, one of the greatest advantages of organoids is that they preserve cell-cell interactions in their 3D organization (**Figure 1**). This is critical, as much of the effort in the hPSC field is dedicated only to making β cells or clusters of β cells, largely ignoring the other pancreatic cell types such as the acinar tissue making up the bulk of the pancreas as well as the other hormone producing cells of the islets such as alpha, delta, and PP cells. The more complex structure of organoids provides an opportunity to study a more complete pancreas-like organ in a dish, importantly examining endocrine-exocrine interactions, which are known to contribute to monogenic forms of diabetes, such as MODY8 (6). While all islet cell types can be generated in 3D cultures using hPSC derived β-like cell differentiations, there is neither organization within the immature β-like cell clusters nor are there normal ratios of endocrine cells in these differentiations. Rather, non-β endocrine cells are a byproduct of the differentiations. Since crosstalk amongst endocrine cell types plays an important role in maintenance of blood glucose homeostasis (74), generating appropriate ratios and organization of endocrine cells as found in healthy islets should improve functionality, which is part of the promise of using organoids rather than hPSC derived β-like differentiations. One breakthrough example of this comes from the identification of a progenitor population in the adult murine islet which can be isolated by the surface marker Procr (69). Wang and colleagues sorted Procr+ cells from adult murine islets to generate islet-like organoids in culture that contained all islet cell types that were functional and reversed diabetes when transplanted into immunocompromised mice. While the existence of an islet progenitor population has long been controversial (75–77), this work demonstrates an exciting advance, although a similar population has not yet been clearly defined in humans.

In addition to improving functionality, the 3D structure of organoids can lend additional insight into the mechanistic analyses performed at intermediate stages of development, like during the critical window of endocrine progenitor specification. As discussed previously, hPSC derived β-like cell differentiations allow for interrogation of these stages using ChIP-, RNA-, scRNA-, and ATAC-seq. However, by generating a 3D structure, organoids more closely mimic normal developmental processes. Exciting recent work has indeed demonstrated this by comparing pancreatic progenitors derived as organoids or 2D hPSC cultures with *in vivo* pancreatic progenitors using whole genome sequencing techniques (78). The organoid samples more closely recapitulated endogenous pancreatic progenitor gene expression and became more “pancreas-like” by excluding non-pancreas genes during their differentiations as compared to more traditional 2D β-like cell differentiations. If the goal is to model true human development in a dish, organoids hold great promise.

While great strides have been made in generating pancreatic organoids, there are still several caveats to this model. One potential pitfall is that similar to the hPSC derived β cell field, there is no standard protocol across all labs working on these systems. Many reports demonstrate high reproducibility within the same research group (78, 79), but comparisons between the different pancreatic organoid protocols have yet to be completed. It is likely that differences will exist since each protocol uses a variety of methods to generate a 3D structure. Many protocols use Matrigel to help generate this architecture, but others use biomaterials and encapsulation (80). Therefore, in addition to mapping all the signals required at each step of the differentiations, we also need to carefully consider how the cells are cultured, as cell-cell interactions are a key determinant of organoid formation and function.

While there has been a substantial role identified for GATA6 in pancreatic cancer by using organoid models of this disease (81, 82), there have been a paucity of studies examining mechanisms of GATA6 action during islet development or function using either hPSC- or primary organ-derived organoids. This presents an exciting opportunity to use recent protocols (78, 79) to generate pancreatic organoids to shed light on the discrepancies between mouse and human GATA6 function. As previously mentioned, the role of GATA6 during endocrine progenitor specification remains unclear: in mice, GATA4 compensates for loss of GATA6 (34, 35), whereas during hPSC derived β cell differentiations GATA6 inhibition leads to downregulation of GATA4, preventing compensation (50). Even within hPSC derived β-like cell differentiations there are discrepancies. The Vallier group found reduced definitive endoderm differentiation upon inducing patient specific GATA6 mutations in their hPSC lines, whereas both the Huangfu and Gadue groups did not observe similar deficiencies in analogous experiments. These results could be explained by technical differences between protocols or by the specific mutations being modeled, but they could also be indicative of the absence of cell-cell or tissue interactions when using hPSC derived β cell differentiations as a model. The 3D structure of organoids that generates multiple cell types could provide substantial insight into these differences by allowing for better cell-cell communication. Organoids also provide an exciting means to examine inter-organ crosstalk, as the cardiac mesoderm sends signals like FGF and BMP to the pancreas during development and could be co-cultured (either healthy or mutant) with foregut endoderm, which gives rise to pancreas progenitors. While there are still caveats to the organoid system, they provide another opportunity to examine gene function in health and disease.

## Conclusion

The model systems discussed in this review have collectively provided vital information about pancreas development, islet function, and diabetes. They each have unique advantages that allow researchers to address the knowledge gaps plaguing our understanding of the molecular underpinnings of diabetes, but each model has its own disadvantages as well. To better understand how diabetes arises and progresses, a combinatorial approach leveraging the strengths of multiple models must be employed. GATA6 is just a single example, but the principles described in the studies discussed here are broadly applicable to any gene or genes implicated in disease. Understanding the molecular mechanisms of development and disease using the models presented in this article will improve current therapies to treat diabetes and offer the best hope for a cure.

## Author contributions

Conceptualization: DL, LS. Writing: DL, DS, and LS. Supervision: DL, LS. Funding acquisition: DL, DS, and LS. All authors contributed to the article and approved the submitted version.

## Funding

This work was supported by the National Institutes of Health K99-DK128537 (DL); National Institutes of Health F31-DK131769 (DS), and National Institutes of Health R01-DK118155, R01- DK082590, and P30-DK116073 (LS).

## Acknowledgments

We would like to thank members of the Sussel laboratory for helpful discussions throughout the conceptualization, writing, and editing of the manuscript.

## Conflict of interest

The authors declare that the research was conducted in the absence of any commercial or financial relationships that could be construed as a potential conflict of interest.

## Publisher’s note

All claims expressed in this article are solely those of the authors and do not necessarily represent those of their affiliated organizations, or those of the publisher, the editors and the reviewers. Any product that may be evaluated in this article, or claim that may be made by its manufacturer, is not guaranteed or endorsed by the publisher.
